# An Assessment of Heavy Metals Toxicity in Asian Clam,* Corbicula fluminea*, from Mekong River, Pa Sak River, and Lopburi River, Thailand

**DOI:** 10.1155/2019/1615298

**Published:** 2019-07-15

**Authors:** Ai Yin Sow, Koh Han Dee, Seong Wei Lee, Aweng A. L. Eh Rak

**Affiliations:** ^1^Faculty of Agro-Based Industry, Universiti Malaysia Kelantan, Locked Bag No. 100, Jeli Campus, 17600 Jeli, Kelantan, Malaysia; ^2^Faculty of Earth Science, Universiti Malaysia Kelantan, Locked Bag No. 100, Jeli Campus, 17600 Jeli, Kelantan, Malaysia

## Abstract

High population density and economic development attributing to the changes in water quality in Pa Sak River, Lopburi River, and Mekong River have attracted great attention. This research aimed to determine the pollution of heavy metals in collected clams at three different study sites. Bioaccumulation of heavy metals in Asian clam (*Corbicula fluminea*) may be likely to cause serious health effects on human beings. The clams sampled from three different rivers (Mekong, Pa Sak, and Lopburi) from Thailand were analyzed for the presence of heavy metals (Zn, Cu, Cd, Cr, Mn, and Pb) with an air-acetylene flame atomic absorption spectrophotometer (AAS). Among the heavy metals studied, Zn was recorded as having the highest concentration (127.33-163.65 *μ*g/g) among the three rivers. The observed mean concentration of Cu was in the range of 84.61-127.15 *μ*g/g followed by Mn (13.96-100.63 *μ*g/g), Cr (5.79-15.00 *μ*g/g), Pb (3.43-8.55 *μ*g/g), and Cd (0.88-1.95 *μ*g/g). Overall, Asian clam from Pa Sak River was found to contain high concentrations of Zn, Cu, Cd, Cr, and Pb compared to Mekong and Lopburi River.

## 1. Introduction

Water pollution is a serious environmental problem in Thailand (Kornprabha et al., 2005) and Cambodia (Viet et al., 2016) with increased growth in population, industry, and agriculture (Molle, 2002). Anthropogenic pollutants from residential areas include surfactants, pharmaceuticals, and insect repellent as well as agricultural chemicals like pesticides and industrial chemicals such as inorganics and heavy metals (Knepper et al., 1999). Heavy metals can enter into the water supply through man-made pollution sources which include mining, metal smelting and processing, industrial wastewater, fossil fuels burning, application of fertilizers, pesticides, and household garbage as well as natural sources [1]. According to Salomons [2], the bioavailability of metals in the geological matrix reaches equilibrium based on several physicochemical parameters such as pH, temperature, potential redox, and organic content. Particular pollutants such as heavy metals (Cd and Pb) can accumulate in shellfish through their diet and respiration and incorporate the metals within their tissues, resulting in the seafood contamination which could pose risks to human health (Yap et al., 2008).

*Corbicula fluminea*, also known as Asian clam, is one of the most native freshwater species in the family Corbiculidae in Malaysia. This species of clam which is widely distributed in the freshwater bodies is easy to collect and can be used as excellent bioindicator to monitor heavy metals pollution in freshwater ecosystems (Liang et al., 2004). On other note, Asian clam is a sedimentary filter feeder and has shown high capability to bioaccumulate chemical substances dissolved in the water [3, 4] or bound to suspended particles [5]. Filter feeders feed on suspended particles in the water column and are highly exposed to large quantities of contaminants, such as a variety of toxic metals which are derived from anthropogenic activities [6]. These toxic metals from industrialization, agricultural land runoff, oil and gas productions, and tourism development reach water bodies through heavy rainfall which results in the deposition of a high volume of pollutants [7–9]. Moreover, due to the high filtration rate [10] of* C. fluminea*, this clam is able to increase the water clarity by filtering between twenty and one hundred litres of water a day [11].

The use of the total soft tissues of Asian clam for biomonitoring studies of heavy metal pollution has been focused on rather than the clam shells as a quantitative indicator to reflect the heavy metals contamination at the particular coastal area [12]. Baby et al. [13] and Juncharoenwongsa et al. [14] reported that concentrations of Cd, Cr, Cu, Fe, Ni, Zn, and Pb were found in Asian clam and Zn had the highest concentration accumulated in soft tissues. Because of the strong capabilities to bioaccumulate metals, in vivo experiments are often adopted as an acute toxicity test [15].

For the past few decades, the risk assessment of aquaculture production to human health became a pressing topic. Bivalves have been extensively studied with regard to the exposure to heavy metals in marsh clam (*Polymesoda expansa*) and green mussel (*Perna viridis*) [11, 16]. The Asian clam* C. fluminea* is consumed by the majority of the population in Thailand and Cambodia while some do get exported to other countries especially Malaysia. Hence, analyzing the concentrations of heavy metals in Asian calm can assist in monitoring water quality and evaluating the potential risks to consumers caused by consuming polluted seafood. Therefore, this study aimed to determine the level of heavy metals in Asian clams collected from Thailand and Cambodia.

## 2. Material and Method

The collection of Asian clam,* C. fluminea*, was conducted in three different sites, namely, Pa Sak River (14°21'51”N, 100°34'48”N), Lopburi River (14°52'38”N, 100°24'40”E), and Mekong River (33°42.5'N, 94°41.7'E). Pa Sak River which is located in the central of Thailand joins the Lopburi River before they run into Chao Phraya River. According to Nuanchan [17], Pa Sak River and Lopburi River are enriched with faecal coliform bacteria (FCB) concentration and low concentration of DO which lead to the water quality degradation. Mekong River is an important international river, which flows through 6 countries, namely, China, Myanmar, Laos, Thailand, Cambodia, and Vietnam, before it empties into the South China Sea. Since the population and economic development increased dramatically, the source of water had been polluted [18, 19] especially at the Upper Mekong, especially the dams constructed or under construction, which is attributed to the increase in amount and composition of sediment [20–23].

Since the rivers are deep and high tide was observed, the collected clams were obtained directly from the fisherman and no collection of water and sediment was done. The collection of the samples was conducted during the dry season for three different sites. The samples were directly placed into polyethylene bottles upon sampling from the fishermen. However, the samples were rinsed several times with distilled water to get rid of mud and dirt prior to performing the analyses. Process of measuring the initial weight and initial length linear distance from posterior end to anterior end was performed using a digital calliper 0.01 mm and analytical balance 0.1mg precision. The collected clams sizes ranged from 13.0 to 15.0 mm in length and 1.201 to 1.532g in body weight (BW).

Upon cleaning, the clams were placed in folded aluminium foil and dried in an air-circulating oven for 3-4 days at 60°C until a constant dry weight was achieved prior to metal analysis. In this study, the whole dry tissues of clam were used for acid digestion. The dried powdered samples were weighed and placed into a digestion tube with the addition of 10ml of concentrated nitric acid (HNO_3_) (AnalaR grade, BDH 69 %). This reaction was performed at room temperature. Three replicates were digested for each location in this study. Next, the tubes were placed in a hot-digester apparatus at 40°C for 1 hour, after which the temperature was gradually increased up to 140°C for at least 3 hours. Upon completing the digestion procedure, distilled water was added into the tubes up to 40 ml followed by filtration using Whatman No. 1 (Filter speed: medium) filter paper in a funnel. The filtered solution was then collected in an acid-washed polyethylene bottle (Yap et al., 2002a; 2002b).

Following filtration, the collected solution of each sample was tested for the presence of Zn, Cu. Cd, Cr, Mn, and Pb by using an air-acetylene flame atomic absorption spectrophotometer (AAS), Perkin-Elmer Model analyst 900F. The data is presented in *μ*g/g dry weight (dw). Multiple-level calibration standards were employed to generate calibration curves against which sample concentrations were estimated. To ensure the accuracy of the results, the r^2^ of the calibration curve was maintained in the range of 0.998-1.000. In order to confirm the validation of the method, SRM 2976 provided by National Institute of Standards and Technology (NIST) was used for clam samples. The analytical results for the investigated heavy metals in the reference material were within the certified values as follows: Zn (102.70%), Cu (99.98%), Cd (96.34%), Pb (99.30%), Mn (101.24%), and Cr (100.14%).

## 3. Result

Table 1 lists the concentrations of Zn, Cu, Cd, Cr, Mn, and Pb in Asian clams collected from three different locations within Thailand and Cambodia. The concentration of Zn was estimated in the range of 127.33-163.65 *μ*g/g, Cu 84.61-127.15 *μ*g/g, Mn 13.96-100.63 *μ*g/g, Cr 5.79-15 *μ*g/g, Pb 3.43-8.55 *μ*g/g, and Cd 0.88 -1.95 *μ*g/g. The concentrations of Zn, Cr, and Pb were nonsignificant (p>0.05) among the three locations, while the Cd was significantly (p<0.05) different for clam tissues from the three locations. As for Cu, clams from Lopburi and Mekong displayed insignificance (p>0.05) while clams from Pa Sak Lake indicated significant (p<0.05) differences. Based on the overall findings, Asian clam from Pa Sak Lake was found to contain high concentrations of Zn, Cu, Cd, Cr, and Pb compared to Mekong and Lopburi River. However, Mn metal was detected to be of the highest concentration in the Asian clams collected from Mekong River. Nevertheless, insignificant (p>0.05) difference was observed for Mn in clams from the Mekong and Pa Sak River compared to Lopburi which was significant (p<0.05). On the other hand, Table 2 postulated that the Pa Sak River was the highest polluted water body, followed by Mekong and Lopburi River based on the descending order of heavy metals according to the localities.

## 4. Discussion

The results revealed that the Asian clams of Pa Sak River accumulated high concentrations of Zn, Cu, Cd, Cr, Mn, and Pb compared to the clams in Mekong and Lopburi River. Among the metals tested, Zn was present in high concentrations in the Asian clams from Pak Sak River, suggesting that this metal is an essential element for most of the bioactivities within the clam. Similarly, high concentrations of Zn were observed in marsh clam and green mussel studies conducted by Harsono et al. [11] in the Mengkabong River, Sabah. Additionally, Jaffar and Pervaiz (1989) declared that the accumulation of high Zn could be related to specific metabolic processes and coenzyme-catalysed reactions involving Zn within fish. Nevertheless, the estimated Zn level (127.33-163.65 *μ*g/g) in this study was lower than the concentrations reported in samples collected from Kg. Pasir Putih, Johor [24], and Mengkabong River, Sabah [11]: 368.0 *μ*g/g and 377.1 *μ*g/g, respectively. Furthermore, the contents of Zn in clam tissues collected from all three study areas were below the permissible limit set by the Ministry of Public Health, Thailand, which is 667.00*μ*g/g dw (MPHT, 1986).

Cu is another essential element required for enzymatic systems (Kanakaraju et al., 2008). Cu was found to be nonsignificant (p>0.05) in clams from the Lopburi and Mekong River (Table 1). However, the Cu concentrations recorded in this study were slightly higher in samples collected from Tanjung Lumpur, Pahang (15.5 ±3.09 *μ*g/g) [16]. The high concentrations of Cu in the Pa Sak, Lopburi, and Mekong River could be due to the fact that these are busy routes used for boating and tourism activities. Hence, the deposition of fuelling may have resulted in the increased levels of Cu in these areas and subsequently accumulated in the Asian clams in the respective rivers. In addition, the increasing demand for agricultural products in the Chao Phraya basin has resulted in the rapid use of pesticides and fertilizers. The toxins from the excessive use of pesticides and fertilizers may have been discharged as runoff from agricultural areas into the surface water causing contamination (Kruawal et al., 2005). Although the concentration of Cu was found to be high, it is still well below the limits set by the Ministry of Public Health, Thailand, at 133.00 *μ*g/g dw. However, Cu may pose a serious threat to the aquatic organisms if the permissible limit is exceeded.

Next, Cd is a nonessential element which is considered toxic for both vertebrates and invertebrates alike (Elinder 1982; Taylor, 1983). The mean concentration of Cd recorded for all three locations ranged between 0.88 and 1.95 *μ*g/g, where the peak value was recorded in Asian clams collected from Thailand's Pa Sak River. Although the latter is still lower than the concentration of Cd recorded in samples collected from Parit Jawa, Johor, 3.59 *μ*g/g [24], it has breached the permissible limits set by the Ministry of Public Health, Thailand, and Malaysian Food Regulation at 1.00 *μ*g/g. Hence, it is strongly suggested that the consumption of clams from these locations for a longer period of time may cause a serious health threat to the local consumers.

Cr in the soft tissues of clams in this study ranged from 5.79 to 15.0 *μ*g/g for all study areas, which is lower than the concentrations reported in Northwest Sabah (20.2 *μ*g/g) [11]. As a nonessential metal, Cr is an element required in small quantity for the biological processes of organisms where its permissible limit is 3 to 5 mg/kg as stated by Popa (2006), as short exposure to Cr can irritate the skin and cause ulceration and long term exposure may be related to serious health hazards effects such as mutation and damage to cells, circulatory and nerve tissues, kidney, and liver [25].

Sediment is the sink of various contaminants including heavy metals [26]. Mekong River was recorded as the second highest polluted after Pa Sak River. This was observed from the high concentrations of metals in clams collected from this area, especially of Mn (100.63 ±0.54) (Table 1), compared to the other two sites. This value was also higher than the data recorded by Xu et al. (2016) in oysters, ranging from 23.8 to 65.0 *μ*g/g dw. High concentrations of Mn were also detected in the soft tissues of bivalves in previous studies conducted in Mandori area at 21.6-140.2 *μ*g/g, Chapora area at 10.8-98.3 *μ*g/g (Al-Usmani et al., 2015), and Mengkabong River, Sabah, at 91.73 ± 13.1 *μ*g/g [11]. The bioaccumulation of high levels of Mn in whole tissues of clam represents its bioavailability in the water and sediments. Besides, Ferreira et al. [27] stated that the spatial variation of heavy metals accumulation in bivalves from different sites is affected by anthropogenic activities. Furthermore, several other factors affecting the accumulation of heavy metals in bivalves include species, age, feeding habits, and reproductive as well as physiochemical state [28]. Since Asian clam is a sedimentary organism and is usually buried in the sediment, this might explain the high levels of Mn in its soft tissues particularly due to the nature of the study area (Al-Usmani et al., 2015). According to WHO (1993), the permissible limit for Mn in molluscs is 100 *μ*g/g.

On the other hand, Pb is a nonessential metal known to cause toxic effects even at low levels. The Pb levels recorded in this study are higher than that of a previous study conducted by Ong et al. [29]. The Asian clams from Pa Sak River accumulated Pb above the permissible limit set by the Ministry of Public Health, Thailand (1986), at 6.67 *μ*g/g dw. However, the levels of Pb in clams from the Mekong River and Lopburi River were within the safe range. The deposition of Pb in Asian clams from Pa Sak River might have been due to the burning of fossil fuels during boating activities [30]. Mansour et al. [31] reported that the increasing Pb load is attributed to the tourism activities in the Hurghada area, Egypt. Furthermore, Pb tends to deposit and bioaccumulate in the younger bivalve due to their higher metabolism compared to those of bigger size [32]. This poses a health threat to the local consumers.

Based on the CGIAR (2018), pollution hotspots in Thailand included the Lopburi River and Pa Sak River (Sunthorn, 2010). Sunthorn (2010) also reported that the Pa Sak and Lopburi River passing Ayutthaya province were polluted causing the water to be dark green in color and emitting foul smell. The residential areas along the Pa Sak River could have also contributed towards the heavy metals loading in this river. Furthermore, agricultural, industrial, and tourism activities along the Pa Sak River may have also contributed to the emission of large quantities of metal toxicants into the freshwater environment. Therefore, these factors, in general, have collectively accounted for the high concentrations of Zn, Cu, Cd, Cr, and Pb in the Asian clams collected from Pa Sak River compared to that of Lopburi and Mekong Rivers. Since Lopburi River joins the Chao Phraya together with Pa Sak River, no significant variations were observed in the accumulation of heavy metals in the Asian clams collected from these two sites.

## 5. Conclusion

In conclusion, the accumulation of Zn and Cu concentrations in clams from the three study areas was well below the limits proposed by the Ministry of Public Health, Thailand (1986). However, Asian clams collected from Pa Sak River exhibited higher Pb, Cd, and Cr metals, whereas only high biomagnification levels of Cr metal were found in clams from Lopburi River. As for Mekong River, elevated levels of Cd, Cr, and Mn were found in the clams. Metals such as Pb, Cd, Cr, and Mn can biomagnification into the food web and, thus, may exert toxicological health hazards to humans. In short, high population density and rapid economic development have resulted in the loading of anthropogenic pollutants in water bodies, thus affecting water function and health of the ecosystem (Chris et al., 2006; He et al. 2006). Hence, since the study areas are part of the fishing ground, regular monitoring of heavy metals in aquatic organisms should be conducted periodically.

## Figures and Tables

**Table 1:** Mean concentrations of Zn, Cu, Cd, Cr, Mn, and Pb (*μ*g/g ± SD) in freshwater Asian clam.

Metal						
Location	Zn	Cu	Cd	Cr	Mn	Pb
Lopburi River	157.73±39.00^a^	88.55±9.75^b^	0.88±0.08^c^	6.95±2.10^a^	13.96±2.55^b^	3.43±2.76^a^
Mekong River	127.33±29.09^a^	84.61±2.95^b^	1.72±0^b^	5.79±1.51^a^	100.63±5.40^a^	3.61±1.50^a^
Pa Sak River	163.65±69.93^a^	127.15±0.91^a^	1.95±0.07^a^	15.0±8.63^a^	95.2±13.29^a^	8.55±9.55^a^

Remark: *post hoc*: mean metal concentrations of different location sharing a common letter for a particular metal present no significant differences, *p>0.05; *having different letters represents a significant difference, p<0.05.

**Table 2:** Heavy metals concentration of *C. fluminea *in descending order from three rivers.

Metal	The sequence in descending order
Zn	Pa Sak >Lopburi>Mekong
Cu	Pa Sak >Lopburi>Mekong
Cd	Pa Sak >Mekong>Lopburi
Cr	Pa Sak >Lopburi>Mekong
Mn	Mekong >Pa Sak >Lopburi
Pb	Pa Sak > Mekong>Lopburi

## Data Availability

The data used to support the findings of this study are available from the corresponding author upon request.
